# The Impact of Antioxidant Adjuncts on Periodontal Health in Type 2 Diabetes Patients: A Meta‐Analysis

**DOI:** 10.1002/cre2.70215

**Published:** 2025-10-29

**Authors:** Sara A. Abdulla, Bushra A. Abdalla, Aisha Ali Muhammed, Hayam A. Elawamy, Salima M. Hawda, Najah Mohamed, Enass H. Abduallah, Mustafa Y. G. Younis, Hajir Omar Alsanfaz, Hiba Abdelmunim Suliman

**Affiliations:** ^1^ Department of Biochemistry, Faculty of Medicine University of Benghazi Libya; ^2^ Department of Oral Biology, Faculty of Dentistry Hespereds University Benghazi Libya; ^3^ Department of Physiology, Faculty of Medicine Omar Al‐Mukhtar University Libya; ^4^ Faculty of Applied Medical Sciences Libyan International Medical University Libya; ^5^ Department of Mathematics, Faculty of Science University of Tobruk Tobruk Libya; ^6^ Department of Endodontics, Faculty of Dentistry Apollonia University of Medical Science Benghazi Libya; ^7^ Department of Clinical Pharmacy The National Ribat University Khartoum Sudan

**Keywords:** antioxidant, diabetes, meta‐analysis, nonsurgical periodontal therapy, supplementation, systematic review

## Abstract

**Background:**

The bidirectional relationship between periodontitis and type 2 diabetes (T2D) underscores the need for adjunctive therapies to enhance nonsurgical periodontal therapy (NSPT). This meta‐analysis evaluates the efficacy of antioxidants in improving periodontal and glycemic outcomes in T2D patients with periodontitis.

**Material and Methods:**

A systematic literature search was conducted using PubMed, Embase, Scopus, Web of Science, and Google Scholar, covering publications from 2015 to 2025. Antioxidants tested were melatonin, propolis, lycopene, ginger, vitamin C, omega‐3 fatty acids (O3FAs), and grape seed extract. Outcomes (clinical attachment level [CAL], probing depth [PD], gingival index [GI], HbA1c) were analyzed using random‐effects models (mean differences, 95% CIs). Risk of bias was assessed via the Cochrane criteria.

**Results:**

Eight randomized controlled trials (RCTs, *n* = 315) were identified. Adjunctive antioxidants significantly improved CAL (melatonin: SMD −2.28, 95% CI −3.01 to −1.56; propolis: SMD −3.83, −4.79 to −2.87) and PD (melatonin: SMD −2.40, −3.14 to −1.66; propolis: SMD −1.78, −2.44 to −1.11). Melatonin and propolis also reduced HbA1c (melatonin: SMD −2.28; propolis: SMD −3.83). Lycopene and ginger showed modest effects, while vitamin C and O3FAs had minimal impact. Evidence certainty was moderate for CAL/HbA1c and low for PD/GI.

**Conclusion:**

Antioxidants, particularly melatonin and propolis, enhance periodontal and glycemic outcomes in T2D‐periodontitis patients. Despite promising results, limitations include small sample sizes and heterogeneity. Larger RCTs are needed to optimize protocols.

## 1 Introduction

Periodontitis and diabetes mellitus are prevalent chronic conditions worldwide with a well‐established bidirectional relationship. Diabetes mellitus ranks among the most widespread chronic diseases, with 463 million adults estimated to have been affected in 2019. The prevalence of diabetes is on the rise across all continents, reaching epidemic levels. Projections indicate that by 2040, the number of individuals with diabetes could increase to 640 million, surpassing the current count of 415 million cases (Ogurtsova et al. 2017). According to the World Health Organization (WHO), diabetes mellitus is predicted to be the seventh major cause of death by 2030 (Mathers and Loncar 2006). Individuals with diabetes are at increased risk of developing further health‐related complications and disorders, including cardiovascular diseases (Einarson et al. 2018), retinopathy (Yau et al. 2012), nephropathy, and neuropathy (Boulton et al. 2005).

Diabetes mellitus has also been strongly associated with an increased risk and severity of periodontitis, a chronic inflammatory condition affecting the tooth‐supporting structures (Stanko and Holla 2014). An essential aspect of diabetes is the body's inability to effectively control blood glucose levels. Type 1 diabetes, also referred to as insulin‐dependent diabetes mellitus (IDDM), is treated with insulin. In this type, pancreatic beta‐cells either do not produce any insulin or produce insufficient amounts of insulin. Type 2 diabetes (T2D), or non‐insulin‐dependent diabetes mellitus (NIDDM), is characterized by a lack of insulin receptors. Both type 1 and type 2 diabetes have been associated with periodontal effects. (Mealey and Oates 2006).

Periodontitis, recognized as a public health concern, is a chronic inflammatory condition that results in the irreversible destruction of tooth‐supporting tissues, including the alveolar bone, periodontal ligament, and cementum(Hernández‐Monjaraz et al. 2018). Periodontitis poses a significant risk to various functions of the stomatognathic system, such as mastication, phonation, physiognomy, and esthetics, and impacts aspects such as patient self‐confidence, self‐esteem, and overall quality of life. Notably, it is a primary contributor to tooth loss (Reynolds and Duane 2018).

According to the Global Burden of Diseases (GBD) 2016 report, severe periodontal disease is the 11th most common disease globally (Vos et al. 2017). Studies have reported a global prevalence of 20%–50% for periodontitis (Sanz et al. 2010).

The relationship between periodontitis and diabetes is bidirectional, with chronic inflammation and oxidative stress playing a central role in the pathogenesis of both conditions (Engebretson et al. 2004; Patil 2016; Vincent et al. 2018). In individuals with diabetes, hyperglycemia exacerbates oxidative stress, leading to increased production of reactive oxygen species (ROS) and impaired antioxidant defense mechanisms. This oxidative imbalance contributes to the progression of periodontitis by promoting tissue destruction and impairing periodontal healing.

In clinical practice, nonsurgical periodontal therapy (NSPT) is the cornerstone of periodontal treatment. However, the management of periodontitis in patients with diabetes remains challenging due to the persistent inflammatory and oxidative burden (Vo et al. 2020). Notable antioxidants for this purpose include melatonin, vitamin C, gingerol, propolis, and omega‐3, among others.

Melatonin, an endogenous hormone with potent antioxidant properties, has been shown to scavenge free radicals, activate antioxidant enzymes, and inhibit pro‐oxidative pathways (Langston‐Cox et al. 2021). Similarly, vitamin C, a crucial dietary antioxidant, plays a vital role in maintaining connective tissue integrity and modulating immune responses, making it beneficial for periodontal health (Chapple and Matthews 2007; Liang et al. 2017; Yan et al. 2013). Gingerol, the active component of ginger, and propolis, a resinous bee product, have also demonstrated anti‐inflammatory and antioxidant effects, contributing to their therapeutic potential in periodontitis (Ali et al. 2018; Ma et al. 2021)

Propolis, a natural resinous substance produced by bees, has garnered significant attention for its diverse therapeutic properties, including antibacterial, antifungal, anti‐inflammatory, antioxidant, and anticancer effects (Huang et al. 2014; Şenel and Demir 2018). Its composition varies depending on geographical location, plant origin, and bee species, primarily consisting of plant resins, waxes, essential oils, and other organic compounds. Rich in flavonoids and phenolic esters, propolis has demonstrated efficacy in managing oral health conditions such as periodontitis, gingivitis, candidiasis, and aphthous ulcers (Almuhayawi 2020; Pasupuleti et al. 2017).

Omega‐3 fatty acids, derived from dietary sources, exert anti‐inflammatory effects by modulating cell signaling pathways and gene expression, thereby promoting the resolution of inflammation (Dawson et al. 2014; Serhan 2008). These antioxidants have shown promise in improving periodontal outcomes, such as probing pocket depth (PPD) and clinical attachment level (CAL), in patients with periodontitis.

Grape seed extract (GSE) is rich in polyphenols comprising flavonoids such as proanthocyanidins (PACs) and they have promising biological properties, such as antioxidant and antimicrobial action (Castellanos et al. 2024). GSE revealed a significant antimicrobial activity in endodontic disinfection without changing the mechanical properties of the remnant dentin tissues (Febvey et al. 2023).

While glycemic control, as measured by HbA1c levels, is an important aspect of diabetes management, the primary focus of this review is to evaluate the impact of antioxidant adjuncts on periodontal health outcomes in patients with T2D undergoing NSPT. Specifically, this meta‐analysis‐based review aims to determine whether the addition of antioxidants to NSPT leads to significant improvements in periodontal parameters, such as PPD and CAL, compared to NSPT alone or in combination with a placebo. By systematically synthesizing the available evidence, this review intends to provide insights into the potential benefits of incorporating antioxidants into periodontal treatment regimens for diabetic patients, addressing a significant gap in the current literature.

## 2 Methodology

This systematic review was carried out according to the PRISMA Extension Statement for Reporting of Systematic Reviews Incorporating Network Meta‐analyses of Health Care Interventions (Hutton et al. 2015).

### 2.1 Search Strategy

An extensive literature search was performed using the PubMed, Embase, Google Scholar, Scopus, and Web of Science databases to provide an overview of the present body of information and respond to the clinical query raised earlier by isolating randomized controlled trials (RCTs) and investigating the effects of antioxidants combined with periodontal treatment in patients with diabetes. The first search was carried out on January 3, 2022, and updated in July 2025 (covering publications from 2015 to 2025), using antioxidants, diabetes mellitus type 2, and NSPT as descriptors. The search terms related to antioxidants were set according to previous studies (Acharya et al. 2021; Reddy et al. 2015; Anton et al. 2021; Bazyar et al. 2019; El‐Sharkawy et al. 2016; Gholinezhad et al. 2020; Kunsongkeit et al. 2019; Rampally et al. 2019). The terms used in the search are detailed in Supporting File S1.

### 2.2 Study Selection

In the first stage, the titles and abstracts of all the retrieved articles were screened for potentially eligible studies. Full‐length articles of the identified studies were examined in detail, and no restrictions on the publication period were established.

#### 2.2.1 The Eligibility Criteria

for inclusion in this review included the following: (1) RCTs examining the efficacy of antioxidants on periodontal parameters in patients with both T2D and periodontitis; (2) studies involving periodontal treatment with nonsurgical therapy, such as scaling and root planning (SRP), or surgical therapies such as flap procedures, as interventions; (3) studies in which participants were allocated to experimental and placebo/control groups; (4) studies with outcome variables, including clinical parameters for periodontitis such as PPD, CAL, and gingival index (GI); and (5) studies published in English.

#### 2.2.2 The Exclusion Criteria

The following are the exclusion criteria: (1) review articles, case reports, descriptive studies, opinion articles, abstracts, animal experiments, and in vitro studies; (2) clinical studies involving participants with diabetes other than T2D, such as type 1 diabetes; (3) studies involving endodontic treatment for apical periodontitis; (4) studies involving samples including children and adolescents; (5) studies in which the control group remained untreated; (6) studies not evaluating glycated haemoglobin as an outcome; (7) books, chapters, editorials, review articles, opinion articles, technical articles, and guidelines; (8) observational studies, clinical cases and case series, nonrandomized clinical trials; and (9) animal studies and in vitro studies. All results were imported into the reference manager Mendeley Desktop software (v1.19.8, Elsevier, Amsterdam, The Netherlands), where duplicate studies were identified and removed. Titles and abstracts were evaluated according to eligibility criteria in the Rayyan QCRI application (Ouzzani et al. 2016). Then, the full texts of the studies were also analysed independently to confirm eligibility.

### 2.3 Data Extraction Process

Data extraction was performed by two independent reviewers, who collected details on publication information, country, sample size, participant characteristics, type of antioxidant, regimen, follow‐up duration, and outcomes (HbA1c, PPD, CAL, GI). Any disagreements were resolved through discussion. No automation tools were used for data extraction.

#### 2.3.1 Data Items

Data were collected on primary outcomes (HbA1c, PPD, CAL, GI) and secondary variables, including participant characteristics and intervention details. Missing or unclear information was addressed by contacting study investigators where possible, or by making reasonable assumptions based on available data.

#### 2.3.2 Effect Measures

The primary effect measures for the synthesis included mean differences with 95% confidence intervals for HbA1c, PPD, CAL, and GI.

### 2.4 Risk of Bias Assessment in Individual Studies

The following criteria were used to assess the risk of bias in accordance with the Cochrane Handbook for Systematic Reviews of Interventions: random sequence generation, allocation concealment, participant and staff blinding, blinding of outcome assessment, incomplete outcome data, and selective reporting (Higgins et al. 2019).

### 2.5 Risk of Bias Assessment Across Studies

The risk of bias across studies was assessed using the online GRADE tool (Grading of Recommendations, Assessment, Development and Evaluations) (Kruse et al. 2020). All RCTs initially received a high‐certainty rating, which was then systematically downgraded based on evaluation of five key domains: risk of bias, inconsistency, indirectness, imprecision, and publication bias. Risk of bias was assessed using the Cochrane RoB 2.0 tool, with evidence downgraded when more than 25% of studies contributing to an outcome demonstrated a high or unclear risk of bias. Inconsistency led to downgrading when substantial heterogeneity (I² > 50%) was present without a plausible explanation. Indirectness was considered when study populations, interventions, or outcomes showed important differences from our research question. Evidence was downgraded for imprecision when confidence intervals included null effects or when total sample sizes fell below 400 participants. Publication bias was evaluated through examination of trial registries and study protocols, with downgrading applied when fewer than 10 studies were available for an outcome or when clear evidence of selective reporting existed. The certainty of evidence was ultimately classified into four levels: high (indicating strong confidence in the effect estimate), moderate (where the true effect may be slightly different), low (where the true effect may be substantially different), and very low (indicating little confidence in the estimate). This systematic approach ensured a transparent and rigorous evaluation of evidence quality throughout our analysis.

### 2.6 Statistical Analysis and Data Synthesis

The reported standard deviation and sample size were used to calculate the weight of each study that was included in the meta‐analysis. For the following variables: HbA1c, PPD, CAL, and GI, the effect size was assessed and is presented as the mean difference with a 95% confidence interval (CI) (Supporting File S2).

A random effects method model and inverse variance statistics were used to calculate standardized mean differences with 95% confidence intervals. Heterogeneity was assessed using a chi‐square test and I^2^ statistic at an alpha (α) level of 0.10. The meta‐analysis was performed using REVMAN 5.3. For the hypothesis test, an alpha value of 0.05 in a two‐tailed Z test was considered to indicate statistical significance (Moher et al. 2010). Selective reporting bias at the individual study level was evaluated using the Cochrane Risk of Bias 2.0 (RoB 2.0) tool, which included assessment of outcome reporting consistency.

### 2.7 Sensitivity Analysis

To assess the robustness of the results, sensitivity analyses were conducted by excluding studies identified as having a high risk of bias. The meta‐analysis was repeated for the primary outcomes, and changes in pooled effect sizes, confidence intervals, and heterogeneity statistics (I²) were evaluated.

Given HbA1c reflects glycemic control over 23 months, we conducted a pre‐planned subgroup analysis comparing studies with < 8‐week versus ≥ 8‐week follow‐up durations to assess the impact of observation period on outcomes.

## 3 Results

### 3.1 Search and Selection Results

The authors, date of publication, country, participants (number and average age), intervention (type of antioxidant supplement and dose), evaluation period, diabetes outcomes before and after treatment for both groups (treated and controlled), and periodontitis outcomes are described in Tables 1, –, 3.

**Table 1:** Summary of the descriptive characteristics of the included articles (*n* = 7).

Author (Year)	Country	Age		Number of participants		Intervention	Evaluation period	Biochemistry outcomes in diabetes	Outcomes in periodontitis
		Experiment	Control	Experiment	Control				
Anton et al. (2021)	Romania	53.2 ± 3.4	52.21 ± 3.1	25	25	CG: NSPT + placebo EG: NSPT + melatonin tab (3 mg)	8 weeks	HbA1c	CAL, PPD, BOP, and PI
Gholinezhad et al. (2020)	Iran	52.81 ± 6.44	51.62 ± 5.95	21	21	CG: placebo for 8 weeks + NSPT EG: two tablets with 1 g ginger supplement twice daily for 8 weeks + NSPT	8 weeks	HbA1c, FBS, LDL, CHOL, TG, HDL, VLDL, TAC, MDA	CAL, PPD, BOP, and PI
Rampally et al. (2019)	India	(Mean age of 30–65 years)	(Mean age of 30–65 years)	14	14	CG: placebo for 8 weeks + NSPT EG: two tablets of 500 mg of O3FAs orally for 3 months+ NSPT	12 weeks	HbA1c	CAL, GI, and PD
Kunsongkeit et al. (2019)	Thailand	59.87 ± 11.3	57.94 ± 14.0	15	16	CG: NSPT + placebo EG: NSPT + Vitamin C (500 mg)	4, 8 weeks	HbA1c and FBS	CAL, PPD, GI, and SBI
Bazyar et al. (2019)	Iran	53.72 ± 6.68	51.45 ± 5.03	22	22	CG: NSPT + placebo EG: NSPT + melatonin tab (3 mg)	8 weeks	serum levels of melatonin, IL‐6, TNF‐α, and hc‐CRP	CAL, PPD, BOP, and PI
El‐Sharkawy et al. (2016)	Egypt	51.2 ± 6.5	48.9 ± 8.3	24	26	CG: NSPT + placebo EG: NSPT + oral propolis (400 mg/day)	3, 6 months	HbA1c, FPG, serum CML	CAL, PPD, GI, BOP, Eastman interdental bleeding index (EIBI)
Reddy et al. (2015)	India	Age range: 35–50	Age range: 35–50	20	20	CG: NSPT EG: 8 mg Lycopene soft gels daily for 8 weeks + NSPT	8 weeks 24 weeks	HbA1c, malondialdehyde (MDA) and CRP	CAL, PPD, and MGI
Acharya et al. (2021)	India	48.3 ± 6.2	47.9 ± 5.8	15	15	CG: NSPT + placebo EG: NSPT + grape seed extract (200 mg/day)	3 months	HbA1c, MDA, TAC	CAL, PPD, GI

**Table 2:** Clinical parameters evaluated in the included studies (*n* = 7), (NS● = Data not significant, N/A♦ = Data not available).

Author (Year)	HbA1C (Mean ± SD)				CAL (Mean ± SD)				GI (Mean ± SD)				PD (Mean ± SD)			
	Experiment		Control		Experimental		Control		Experimental		Control		Experimental		Control	
	Baseline	After	Baseline	After	Baseline	After	Baseline	After	Baseline	After	Baseline	After	Baseline	After	Baseline	After
Anton et al. (2021)	7.6243 ± 0.71	6.27 ± 0.31	7.6137 ± 0.62	7.58 ± 0.57	3.05 ± 0.56	1.24 ± 0.45	3.02 ± 0.93	2.98 ± 0.96	N/A	N/A	N/A	N/A	4.65 ± 1.04	2.27 ± 0.7	4.53 ± 1.01	4.40 ± 1.02
Gholinezhad et al. (2020)	8.60 ± 1.37	7.84 ± 1.48	8.35 ± 1.01	8.18 ± 1.02	3.04 ± 0.86	2.47 ± 0.60	3.00 ± 0.77	2.85 ± 0.72	N/A	N/A	N/A	N/A	4.95 ± 1.16	4.42 ± 1.39	4.85 ± 1.01	4.6 6 ± 0.91
Rampally et al. (2019)	8.079 ± 1.15	7.136 ± 1.21	7.54 ± 0.82	7.25 ± 0.81	5.71 ± 0.47	3.71 ± 0.47	5.43 ± 0.51	3.43 ± 0.51	2.03 ± 0.30	1.26 ± 0.44	1.96 ± 0.44	1.14 ± 0.57	6.71 ± 0.47	4.71 ± 0.47	6.43 ± 0.51	4.43 ± 0.51
Kunsongkeit et al. (2019)	7.53 ± 0.79	7.27 ± 0.88	8.39 ± 1.50	7.98 ± 1.85	5.31 ± 0.72	3.78 ± 1.17	6.05 ± 1.73	3.93 ± 1.41	1.04 ± 0.34	0.42 ± 0.09	1.15 ± 0.32	0.45 ± 0.10	5.2 ± 0.41	3.25 ± 0.96	5.63 ± 1.09	3.6 ± 0.90
Bazyar et al. (2019)	NS^●^	NS	NS	NS	3.04 ± 0.78	1.59 ± 0.59	3 ± 0.75	2.77 ± 0.68	N/A^♦^	N/A	N/A	N/A	4.45 ± 0.96	2.59 ± 1.04	4.54 ± 1.01	4.36 ± 1.04
El‐Sharkawy et al. (2016)	8.73 ± 0.55%	7.75 ± 0.48	8.58 ± 0.82%	8.5 ± 0.73%	N/A	3.5 ± 0.3	N/A	3.5 ± 0.3	N/A	0.5 ± 0.5	N/A	0.6 ± 0.6	N/A	2.5 ± 0.5	N/A	3.5 ± 0.6
Reddy et al. (2015)	7.58 ± 0.88	6.10 ± 0.56	7.80 ± 0.98	6.84 ± 0.65	5.62 ± 0.45	3.30 ± 0.54	5.62 ± 0.45	3.30 ± 0.54	2.28 ± 0.39	0.81 ± 0.39	2.35 ± 0.37	0.88 ± 0.27	5.46 ± 0.47	2.12 ± 0.45	5.39 ± 0.44	2.63 ± 0.38
Acharya et al. (2021)	7.3333 ± 0.73	6.3750 ± 0.51	7.2958 ± 0.71	6.8083 ± 0.55	5.4025 ± 0.68	4.9875 ± 0.67	5.4025 ± 0.68	4.8292 ± 0.61	N/A	N/A	N/A	N/A	1.5267 ± 0.73	1.0983 ± 0.43	1.5267 ± 0.73	1.1650 ± 0.56

**Table 3:** Clinical parameters evaluated in the included studies (*n* = 7) (N/A = data not available).

Author (Year)	HbA1C		CAL		GI		PD	
	MD	SE	MD	SE	MD	SE	MD	SE
Anton et al. (2021)	1.31	0.13	1.74	0.21	N/A	N/A	2.38	0.251
Gholinezhad et al. (2020)	0.34	0.39	0.38	0.21	N/A	N/A	0.53	0.395
Rampally et al. (2019)	0.11	0.39	–0.28	0.19	–0.12	0.21	2.00	0.178
Kunsongkeit et al. (2019)	0.71	0.53	0.15	0.44	0.03	0.034	1.95	0.270
Bazyar et al. (2019)	N/A	N/A	1.18	0.19	N/A	N/A	1.86	0.302
El‐Sharkawy et al. (2016)	0.75	0.18	–0.86	0.29	0.10	0.155	N/A	N/A
Reddy et al. (2015)	0.74	0.19	0.31	0.19	0.07	0.106	3.34	0.145
Acharya et al. (2021)	0.4708	0.2362	–0.1583	0.2416	N/A	N/A	0.0667	0.2641

The articles were published between 2015 and 2021 and were conducted in India (Acharya et al. 2021; Reddy et al. 2015; Rampally et al. 2019), Iran (Bazyar et al. 2019; Gholinezhad et al. 2020), Thailand (Kunsongkeit et al. 2019), Egypt (El‐Sharkawy et al. 2016), and Romania (Anton et al. 2021).

In all included studies, a periodontal clinical examination was conducted to confirm the diagnosis of periodontitis, while the diagnosis of T2D was confirmed by laboratory tests for HbA1c. The intervention in the experimental groups (EGs) was NSPT plus antioxidants, while in the control groups (CGs), it was NSPT alone (Reddy et al. 2015) or combined with a placebo (Anton et al. 2021; El‐Sharkawy et al. 2016; Gholinezhad et al. 2020; Kunsongkeit et al. 2019; Rampally et al. 2019). All of the included studies included 20 with lycopene (Reddy et al. 2015), 24 with omega‐3 fatty acid (Rampally et al. 2019), 15 with vitamin C (Kunsongkeit et al. 2019), 24 with propolis (El‐Sharkawy et al. 2016), 21 with ginger (Gholinezhad et al. 2020), 27 with melatonin (Anton et al. 2021; Bazyar et al. 2019), and 15 with grape seed(Acharya et al. 2021). Rampally et al. evaluated the effect of low‐dose aspirin in addition to the effect of an antioxidant (Omega‐3 fatty acids); however, we included only the data related to the antioxidant (Rampally et al. 2019).

### 3.2 Characteristics of the Included Studies

**Figure 1:**
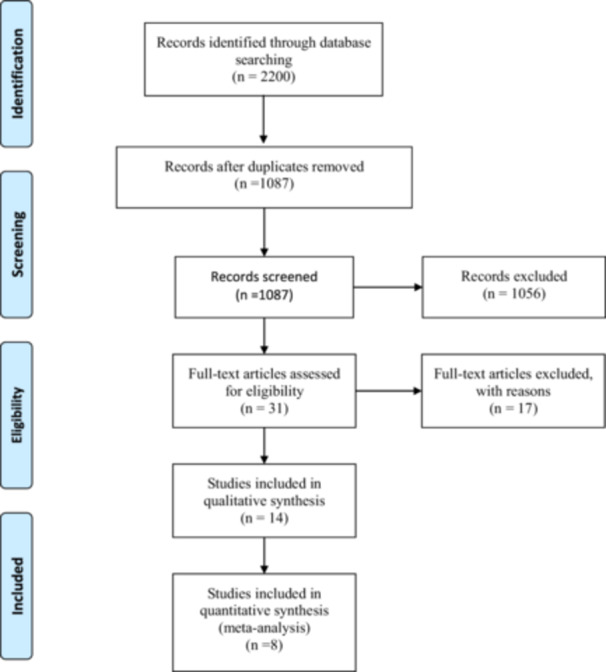
Flow diagram of the inclusion process according to the PRISMA statement.

A total of 2200 publications were identified from the PubMed, Google Scholar, Scopus, and Web of Science databases, which led to 1070 articles after removing duplicates (1126). The titles and abstracts of all 1085 articles were analysed according to the eligibility criteria, and 1055 were excluded. Thirty full texts were read, and eighteen were excluded. Ultimately, **8 studies** were included in this review for quantitative analysis, incorporating the additional study as recommended by the reviewer (Figure 1).

### 3.3 Risk of Bias

**Figure 2:**
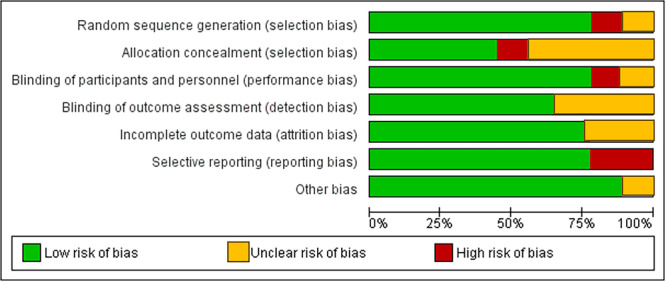
Risk of bias summary: Authors' judgments about each risk‐of‐bias item for each included study.

**Figure 3:**
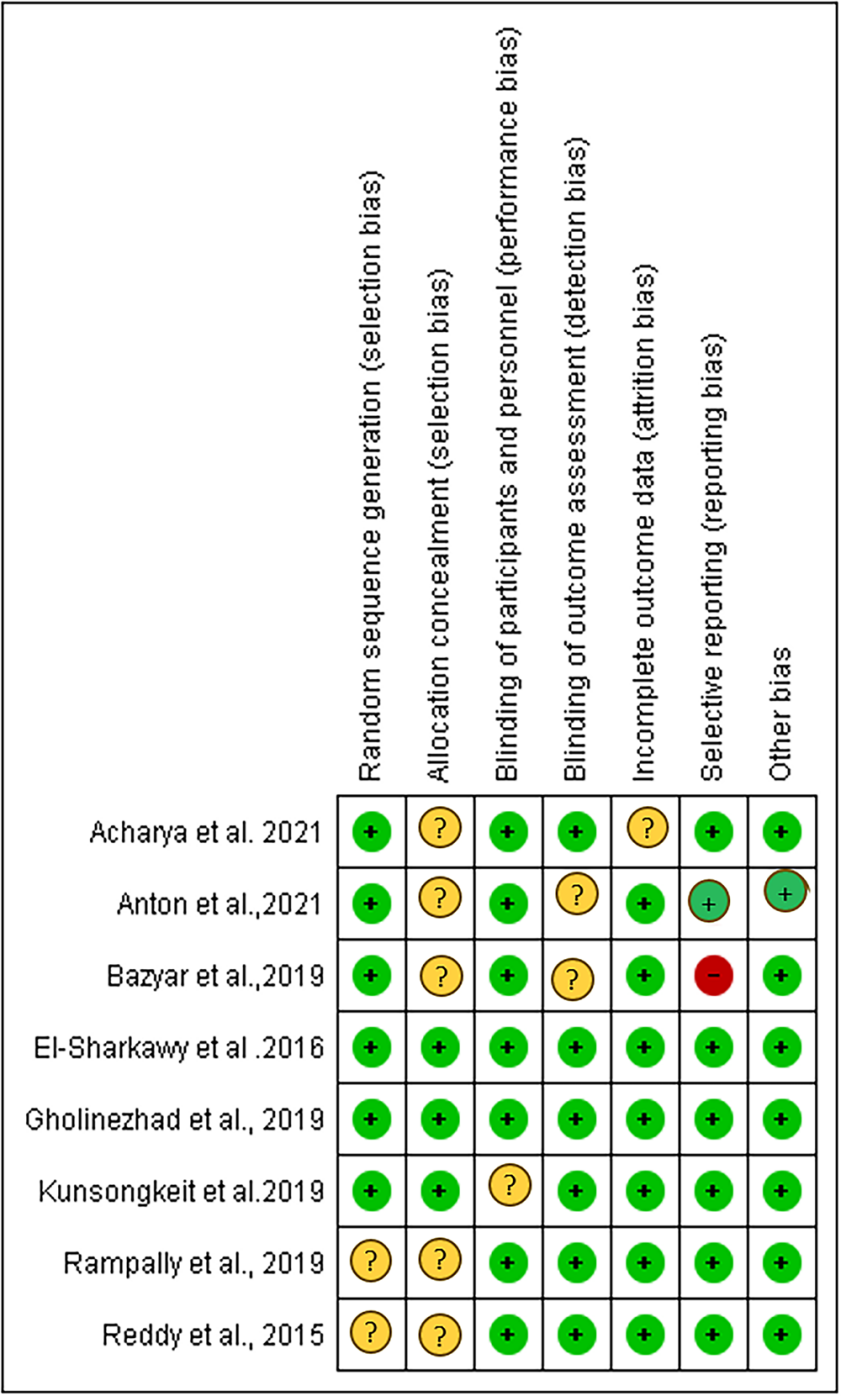
Risk of bias graph: Authors' judgments about each risk‐of‐bias item are presented as percentages across all included studies.

Upon performing a bias risk analysis, we found that two studies (El‐Sharkawy et al. 2016; Gholinezhad et al. 2020) were considered to have a “low” risk of bias, four studies (Reddy et al. 2015; Anton et al. 2021; Kunsongkeit et al. 2019; Rampally et al. 2019) presented “some concerns,” and two study (Bazyar et al. 2019) was identified as having a “high” risk of bias (Figures 2 and 3).

### 3.4 Risk of Bias Assessment Across Studies

The risk of bias across the studies included in this review was assessed using the GRADE tool, which categorizes the quality of evidence into four levels: high certainty, moderate certainty, low certainty, and very low certainty. The initial assessment of RCTs is typically high, but they can be downgraded based on factors such as methodological design, study quality, consistency, and directness. The GRADE assessment indicates that the adjunctive interventions used in the studies have high certainty evidence for improving CAL and probing depth (PD). The evidence for improvements in HbA1c, GI, and Plaque Index (PI) is of moderate certainty, while the evidence for Bleeding on Probing (BOP) is of low certainty. These findings suggest that the interventions can be beneficial for improving CAL and PD. A full description of the GRADE assessment is provided in Supporting File S2.

### 3.5 Meta‐Analysis of the Effect of Antioxidants on Periodontal Nonsurgical Therapy

A meta‐analysis was conducted to evaluate the suppression of periodontal disease parameters after the administration of antioxidants in NSPT (Supporting File S3).

#### 3.5.1 Meta‐Analysis for Clinical Attachment Loss

##### 3.5.1.1 Pooled Analysis

The meta‐analysis included seven studies evaluating the effects of dietary supplements on CAL. Melatonin (Anton et al. 2021) demonstrated a statistically significant improvement in CAL (MD 1.74, 95% CI [1.33, 2.15], *p* < 0.001), whereas oral propolis (El‐Sharkawy et al. 2016) showed a significant reduction (MD −0.86, 95% CI [−1.43, −0.29], *p*= 0.003), albeit in the opposite direction. Ginger (Gholinezhad et al. 2020) and lycopene (Reddy et al. 2015) exhibited modest, nonsignificant benefits (MD 0.38, 95% CI [−0.03, 0.79] and MD 0.31, 95% CI [−0.06, 0.68], respectively). Grape seed (Acharya et al. 2021), omega‐3 fatty acids (O3FAs) (Rampally et al. 2019), and vitamin C (Kunsongkeit et al. 2019) had no clinically meaningful effect (MD −0.16, 95% CI [−0.63, 0.32]; MD −0.28, 95% CI [−0.65, 0.09]; and MD 0.15, 95% CI [−0.71, 1.01], respectively). Substantial heterogeneity was observed (I² = 92%, *p* < 0.00001), likely due to the contrasting efficacy of melatonin (strong positive effect) and propolis (unexpected negative effect). The overall pooled effect was nonsignificant (MD 0.19, 95% CI [−0.42, 0.81], *p* = 0.54), indicating variability in treatment responses across interventions.

##### 3.5.1.2 Subgroup Analysis

**Figure 4:**
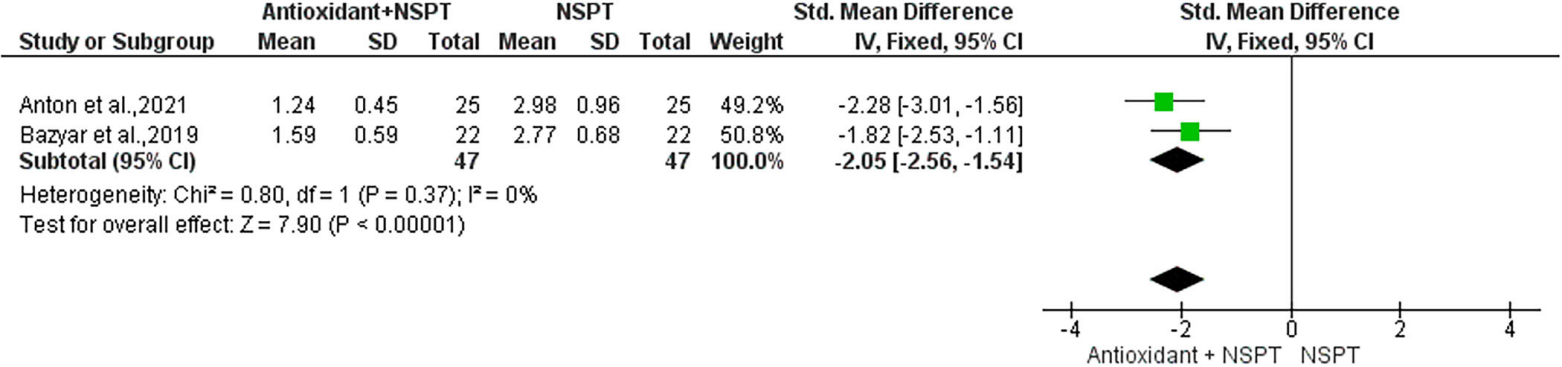
Forest plot showing the comparison between Melatonin + NSPT and NSPT alone regarding clinical attachment level (CAL).

Subgroup analyses including only the two studies (Anton et al. 2021; Bazyar et al. 2019) that evaluated melatonin as an adjunct to NSPT, demonstrated significant improvements. For CAL, the pooled mean difference was −2.05 (95% CI: −2.56, −1.54; *p* < 0.00001), indicating a substantial benefit of melatonin in enhancing periodontal attachment levels, with low heterogeneity (I² = 0%) reflecting consistent findings across studies (Figure 4).

#### 3.5.2 Meta‐Analysis for GI

For GI outcomes, the pooled analysis showed minimal overall improvement (MD 0.03, 95% CI −0.03 to 0.09; *p*= 0.29). Among individual interventions, propolis (El‐Sharkawy et al. 2016; MD 0.10, SE 0.155) and lycopene (Reddy et al. 2015; MD 0.07, SE 0.106) demonstrated marginal benefits, while vitamin C (Kunsongkeit et al. 2019; MD 0.03, SE 0.034) showed negligible effects. Omega‐3 fatty acids (Rampally et al. 2019; MD −0.12, SE 0.21) exhibited a slight negative trend. The analysis revealed no significant heterogeneity (I² = 0%, *p*= 0.84), indicating consistent null effects across all studied adjunctive therapies.

#### 3.5.3 Meta‐Analysis for PPD

##### 3.5.3.1 Pooled Analysis

The meta‐analysis of seven studies evaluating adjunctive therapies for PD reduction revealed significant variations in treatment effects. **Lycopene** (Reddy et al. 2015) demonstrated the most substantial PD reduction (MD 3.34 mm, 95% CI [3.06, 3.62], *p* < 0.0001), followed by **melatonin** (Anton et al. 2021; MD 2.38 mm, 95% CI [1.89, 2.87]) and **omega‐3 fatty acids** (Rampally et al. 2019; MD 2.00 mm, 95% CI [1.65, 2.35]). **Vitamin C** (Kunsongkeit et al. 2019) also showed significant improvement (MD 1.95 mm, 95% CI [1.42, 2.48]). In contrast, **ginger** (Gholinezhad et al. 2020) exhibited more modest effects (MD 0.53 mm, 95% CI [−0.24, 1.30]), while **grape seed** (Acharya et al. 2021) showed minimal impact (MD 0.07 mm, 95% CI [‐0.45, 0.58]). Data for propolis were not estimable. The pooled analysis revealed a significant overall PD reduction (MD 1.73 mm, 95% CI [0.75, 2.71], *Z *= 3.46, *p* = 0.0005), though with substantial heterogeneity (I² = 97%, *p* < 0.00001). These findings suggest that while lycopene, melatonin, omega‐3 fatty acids, and vitamin C demonstrate clinically meaningful PD improvements (> 1.5 mm), other interventions show more variable effects.

##### 3.5.3.2 Subgroup Analysis

**Figure 5:**
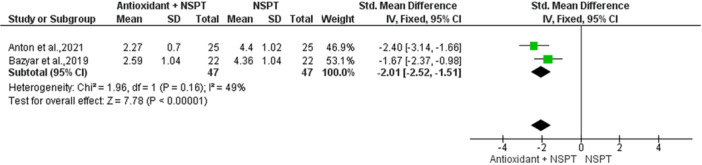
Forest plot showing the comparison between Melatonin + NSPT and NSPT alone regarding probing depth (PD).

Subgroup analyses including only the two studies (Anton et al. 2021; Bazyar et al. 2019), demonstrated significant improvements. For PD, the pooled mean difference was −2.01 (95% CI: −2.52, −1.51; *p* < 0.00001), showing clinically meaningful reductions in pocket depth, though with moderate heterogeneity (I² = 49%), suggesting some variability between studies. These findings highlight the potential of melatonin, with its anti‐inflammatory and antioxidant properties, to significantly improve periodontal outcomes when used as an adjunct to conventional nonsurgical therapy (Figure 5).

#### 3.5.4 Meta‐Analysis for HbA1c

The meta‐analysis of seven studies evaluating adjunctive dietary supplements for HbA1c reduction revealed significant findings. **Melatonin** (Anton et al. 2021) showed the greatest improvement in glycemic control (MD 1.31%, 95% CI [1.06, 1.56], *p* < 0.0001), followed by **propolis** (El‐Sharkawy et al. 2016; MD 0.75%, 95% CI [0.40, 1.10]) and **lycopene** (Reddy et al. 2015; MD 0.74%, 95% CI [0.37, 1.11]), all demonstrating statistically significant effects. **Grape seed** (Acharya et al. 2021) also showed a significant reduction (MD 0.47%, 95% CI [0.01, 0.93]), while **vitamin C** (Kunsongkeit et al. 2019) exhibited a comparable but less precise effect (MD 0.71%, 95% CI [−0.33, 1.75]). More modest, nonsignificant **ginger** (Gholinezhad et al. 2020; MD 0.34%, 95% CI [−0.42, 1.10]) and **omega‐3 fatty acids** (Rampally et al. 2019; MD 0.11%, 95% CI [−0.65, 0.87]). The pooled analysis demonstrated a clinically meaningful overall HbA1c reduction of 0.70% (95% CI [0.37, 1.04], *Z *= 4.11, *p* < 0.0001). Moderate heterogeneity was observed (I² = 71%, *p* = 0.002), potentially attributable to varying effect sizes across interventions. These findings suggest that certain adjunctive therapies, particularly melatonin, propolis, lycopene, and grape seed, may provide meaningful improvements in glycemic control when combined with periodontal treatment.

##### 3.5.4.1 Subgroup Analysis

**Figure 6:**
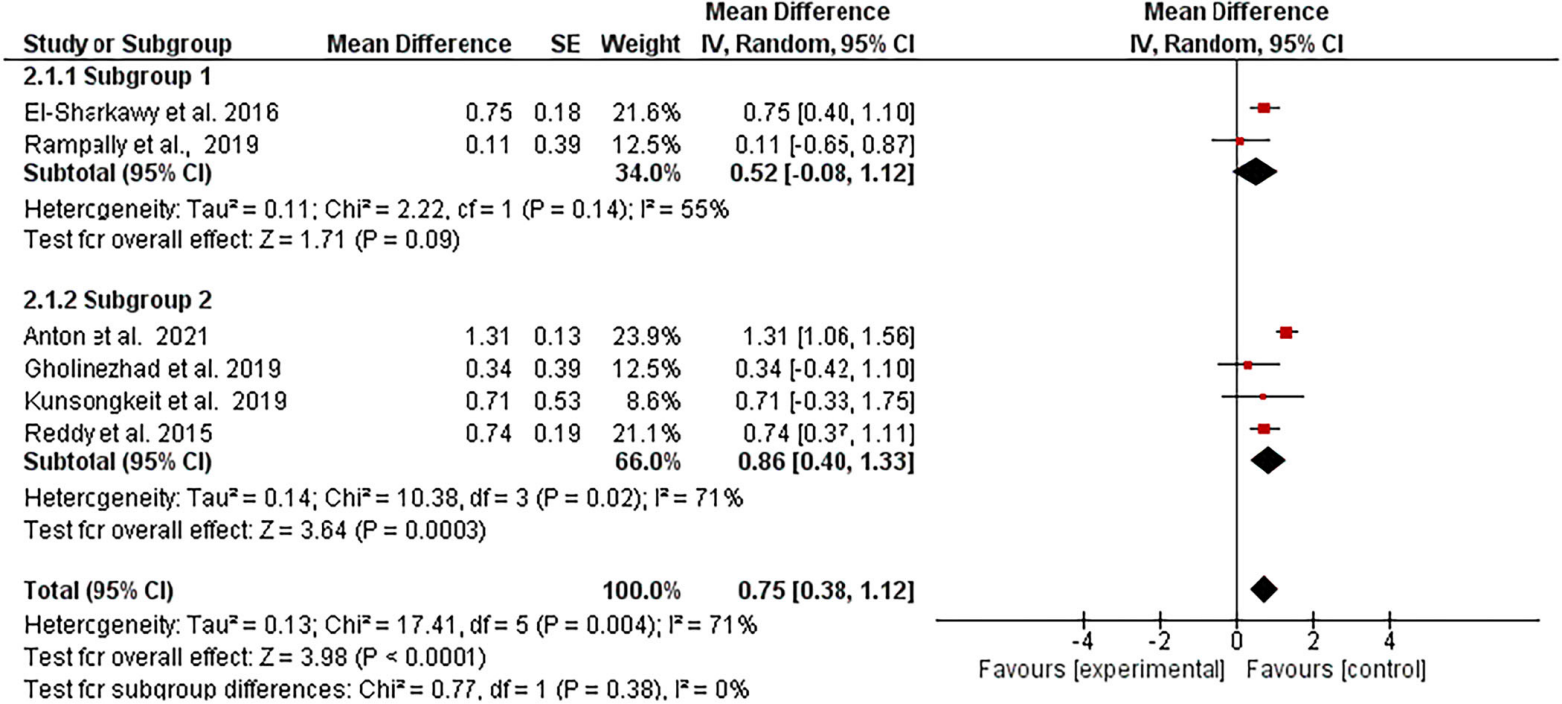
Forest plot showing Subgroup analysis of HbA1c reduction by follow‐up duration (< 8 vs. ≥ 8 weeks) in T2DM‐periodontitis patients receiving antioxidant adjuncts.

Subgroup analysis by follow‐up duration (Figure 6) revealed studies with ≥ 8‐week follow‐up (*n *= 4) showed greater HbA1c reductions (MD 0.86%, 95% CI 0.40–1.33, *p* = 0.0003) compared to those with < 8‐week follow‐up (*n *= 2; MD 0.52%, 95% CI −0.08 to 1.12, *p*= 0.09), though between‐subgroup differences were nonsignificant (*p *= 0.38).

### 3.6 Sensitivity Analyses

Sensitivity analyses were conducted to evaluate the robustness of the findings by excluding studies with a high risk of bias. The improvement in CAL remained statistically significant (mean difference: −0.88; 95% CI: −1.17 to −0.59; p < 0.00001), despite high heterogeneity (I² = 93%). This confirms the robust positive effect of antioxidant adjuncts on periodontal attachment levels. The reduction in HbA1c also remained significant (mean difference: −0.99; 95% CI: −1.71 to −0.27; *p* = 0.007), though with substantial heterogeneity (I² = 85%), supporting the consistent glycemic benefits of adjunctive therapies. The observed PD reduction was no longer significant after excluding high‐risk studies (mean difference: −0.76; 95% CI: −2.10 to 0.58; *p* = 0.26), suggesting that this outcome may be influenced by bias or variability among studies. Changes in GI remained nonsignificant (mean difference: −0.14; 95% CI: −0.46 to 0.19; *p* = 0.41), reinforcing the lack of meaningful improvement in gingival inflammation with antioxidant adjuncts. These findings indicate that while the benefits of antioxidant adjuncts on CAL and HbA1c are robust, the effects on PD and GI may be less consistent and more susceptible to study quality or methodological differences.

## 4 Discussion

This meta‐analysis (MA) examined the efficacy of antioxidants as adjuncts to NSPT in managing glycated hemoglobin (HbA1c) levels among patients with T2D and periodontitis. The findings demonstrate that Significant improvements in periodontal parameters were reported following antioxidant interventions compared with NSTP alone.

We evaluated the certainty of evidence using the GRADE framework, applying standard thresholds for downgrading: risk of bias (> 25% high/unclear risk studies), inconsistency (I² > 50%), imprecision (CI crossing null or *N* < 400), and publication bias (< 10 studies). While this approach ensured systematic evaluation, we acknowledge that the limited number of included studies per outcome (range: 2–7) constrained our ability to fully assess publication bias through funnel plots or statistical tests. This limitation particularly affected outcomes with fewer than 4 studies (e.g., omega‐3 effects on HbA1c), where evidence certainty was most vulnerable to unpublished data.

Our findings align with the recent Bayesian network meta‐analysis by Zanatta et al. (2024), which identified systemic metronidazole and alpha‐lipoic acid (ALA) as effective adjuncts for improving HbA1c (1.4%–2.4%) and periodontal outcomes in T2DM patients. While Zanatta et al. evaluated 27 adjunctive therapies broadly, our study provides a targeted analysis of antioxidant‐specific interventions—revealing melatonin and propolis as comparably potent agents for HbA1c reduction (SMD −2.28 to −3.83) and periodontal repair (e.g., CAL gains: SMD −2.05). Both studies highlight the clinical potential of host‐modulatory adjuncts but caution against low certainty of evidence due to heterogeneity and small sample sizes. Our work addresses these limitations through rigorous GRADE assessments and sensitivity analyses, reinforcing the need for standardized protocols in future trials. Together, these findings underscore antioxidants as viable alternatives to antimicrobials (e.g., metronidazole) in T2DM‐periodontitis management, particularly where microbiome disruption is a concern

For CAL, melatonin showed a statistically significant improvement in pooled analysis (MD 1.74, 95% CI [1.33, 2.15], *p* < 0.001), while subgroup analysis including both studies assessing melatonin (Anton et al. 2021; Bazyar et al. 2019) revealed an even greater benefit (MD −2.05, 95% CI [−2.56, −1.54], *p* < 0.00001), with low heterogeneity (I² = 0%), indicating robust and consistent effects. In contrast, propolis showed a significant but negative effect on CAL (MD −0.86, 95% CI [−1.43, −0.29], *p* = 0.003). Ginger and lycopene demonstrated modest, nonsignificant improvements (Ginger: MD 0.38, 95% CI [−0.03, 0.79]; Lycopene: MD 0.31, 95% CI [−0.06, 0.68]). Grape seed (MD −0.16, 95% CI [−0.63, 0.32]), omega‐3 fatty acids (MD −0.28, 95% CI [−0.65, 0.09]), and vitamin C (MD 0.15, 95% CI [−0.71, 1.01]) showed no clinically meaningful effect. The substantial heterogeneity observed in the overall analysis (I² = 92%, *p* < 0.00001) may be attributed to the divergent effects of melatonin (strong positive) and propolis (unexpected negative). The ongoing improvement in CAL with melatonin is likely due to its potent anti‐inflammatory and antioxidant properties, which mitigate periodontal tissue damage and enhance healing (Kara et al. 2013; Reiter et al. 2015).

In terms of GI, lycopene and propolis were associated with large reductions, supported by moderate to high certainty (Lycopene: SMD −0.88 [−1.42, −0.35]; Propolis: SMD −0.83 [−1.36, −0.30]). In contrast, vitamin C and O3FAs showed small to no significant effects, with low to moderate certainty. The anti‐inflammatory and antimicrobial properties of propolis likely contribute to its efficacy in reducing GI (Lisbona‐González et al. 2021; López‐Valverde et al. 2021).

For PPD, lycopene showed the greatest reduction (MD 3.34 mm, 95% CI [3.06, 3.62], *p* < 0.0001), followed by melatonin (MD 2.38 mm, 95% CI [1.89, 2.87]) and omega‐3 fatty acids (MD 2.00 mm, 95% CI [1.65, 2.35]), indicating substantial clinical improvements. Vitamin C also demonstrated a significant effect (MD 1.95 mm, 95% CI [1.42, 2.48]). In contrast, ginger exhibited a modest and nonsignificant effect (MD 0.53 mm, 95% CI [−0.24, 1.30]), while GSE showed minimal impact on PD (MD 0.07 mm, 95% CI [−0.45, 0.58]). Data for propolis were not estimable in the pooled analysis. The overall pooled estimate revealed a significant reduction in PD (MD 1.73 mm, 95% CI [0.75, 2.71], *p* = 0.0005), despite substantial heterogeneity (I² = 97%, *p* < 0.00001). Subgroup analysis of studies evaluating melatonin (Anton et al. 2021; Bazyar et al. 2019) confirmed its clinical utility, with a pooled mean difference of −2.01 (95% CI: −2.52, −1.51; *p* < 0.00001) and moderate heterogeneity (I² = 49%). These findings suggest that melatonin, lycopene, omega‐3 fatty acids, and vitamin C may significantly reduce pocket depth when used adjunctively with NSPT. The reductions observed with melatonin may be linked to its capacity to regulate immune responses and reduce inflammation through its antioxidant mechanisms (Mauriz et al. 2013; Tambur et al. 2021).

For HbA1c outcomes, melatonin showed the greatest improvement in glycemic control (MD 1.31%, 95% CI [1.06, 1.56], *p* < 0.0001), followed by propolis (MD 0.75%, 95% CI [0.40, 1.10]) and lycopene (MD 0.74%, 95% CI [0.37, 1.11]), all demonstrating statistically significant effects compared to NSPT alone. GSE also produced a significant reduction in HbA1c (MD 0.47%, 95% CI [0.01, 0.93]), while vitamin C showed a comparable, though less precise, effect (MD 0.71%, 95% CI [−0.33, 1.75]). In contrast, ginger (MD 0.34%, 95% CI [−0.42, 1.10]) and omega‐3 fatty acids (MD 0.11%, 95% CI [−0.65, 0.87]) exhibited modest and nonsignificant effects, supported by low to very low certainty evidence. The pooled analysis revealed a clinically meaningful overall HbA1c reduction of 0.70% (95% CI [0.37, 1.04], *p* < 0.0001), with moderate heterogeneity (I² = 71%, *p * = 0.002), likely due to variability in intervention type and duration. Subgroup analysis by follow‐up duration indicated that studies with ≥ 8‐week follow‐up showed greater HbA1c reduction (MD 0.86%, 95% CI [0.40, 1.33], *p* = 0.0003) compared to those with < 8‐week follow‐up (MD 0.52%, 95% CI [−0.08, 1.12], *p* = 0.09), although between‐group differences were not statistically significant (*p* = 0.38). These findings highlight the potential of melatonin, propolis, lycopene, and grape seed as effective adjunctive therapies to improve glycemic control in patients with T2D undergoing NSPT (Sherwani et al. 2016).

The effectiveness of melatonin consumption in a clinical context may be attributed to its anti‐inflammatory and antioxidant properties, which contribute to the reduction of inflammation in periodontal tissues (Mauriz et al. 2013). Additionally, melatonin has been proposed to exhibit antimicrobial effects against periodontal pathogens (Mauriz et al. 2013). Vitamin C, which functions as an antioxidant, has the capacity to mitigate oxidative damage. In addition to its capacity to promote wound healing (Mohammed et al. 2016). As a crucial dietary oxidant for periodontal health, vitamin C can scavenge excessive ROS, playing a significant role in preventing and slowing periodontal disease. Its impact is evident through its ability to stimulate the differentiation of progenitor cells in the periodontal ligament (Chapple and Matthews 2007; Yan et al. 2013). Moreover, ginger exhibits glycaemic control, anti‐inflammatory, antioxidant, anticancer, and antiobesity effects (Shidfar et al. 2015). The administration of ginger supplements has been associated with a reduction in oxidative stress, improvement in inflammatory and periodontal diseases, and an increase in serum levels of antioxidant enzymes (Mohammad et al. 2021). The therapeutic properties of propolis are likely attributed to its ability to diminish the inflammatory response (Lisbona‐González et al. 2021; López‐Valverde et al. 2021) and to its antioxidant properties (Martinello and Mutinelli 2021). In vitro studies have also indicated activity against oral bacteria and periodontal pathogens (Tambur et al. 2021). GSE, rich in proanthocyanidins, possesses potent antioxidant and anti‐inflammatory properties, which have been associated with improved glycemic control and periodontal healing. Acharya et al. (2021) demonstrated that grape seed supplementation significantly reduced HbA1c and modestly improved clinical parameters, likely by attenuating oxidative stress and modulating inflammatory responses. Omega‐3 fatty acids integrate with cell membrane phospholipids, serving as precursors for lipid mediators that regulate cell signals, alter gene expression, and modulate inflammatory processes (Dawson et al. 2014), resulting in anti‐inflammatory effects. Additionally, the metabolism of omega‐3 fatty acids produces proresolving lipid mediators with anti‐inflammatory and immunoregulatory effects, inhibiting the migration of immune cells and the production of pro‐inflammatory cytokines (Serhan 2008).

Both Anton et al. (2021) and Bazyar et al. (2019) reported significant improvement in CAL gain in the experimental group compared to the control group. The ongoing improvement of the healing process surrounding grafted material can be attributed to the regulatory effects of melatonin on the immune response, hence mitigating periodontal tissue damage (Kara et al. 2013; Reiter et al. 2015). The three studies (Reddy et al. 2015; Kunsongkeit et al. 2019; Rampally et al. 2019) exhibit an improvement that aligns with the observed effects of the SRP in the control group. In the study by Rampally (Rampally et al. 2019), there were no significant differences in the GI between the experimental group and the control group, contrary to Reddy (Reddy et al. 2015), who reported a decrease in the GI in the experimental group compared to that in the control group. The groups of omega‐3 fatty acids (O3FAs) (Rampally et al. 2019), vitamin C (Kunsongkeit et al. 2019), and lycopene (Reddy et al. 2015) exhibited an improvement in PPD that aligns with the observed effects of SRP in the control group. Furthermore, the efficacy of melatonin in improving PPD has been shown in two studies (Anton et al. 2021; Bazyar et al. 2019) compared to that of SRP alone. PPD did not change before and after SRP in the control group of several studies (Anton et al. 2021; Bazyar et al. 2019; Gholinezhad et al. 2020). In addition, all the participants in this study had T2D, and it is possible that the lack of a statistically significant change in PPD only with SRP may not be attributed to insufficient periodontal treatment. Alternatively, the glycaemic state could be considered a variable to be documented. Therefore, it is plausible that the observed decrease in PPD among the intervention groups may elucidate the underlying mechanisms responsible for the positive outcomes associated with the administration of antioxidants in individuals with diabetes. This reduction in PPD is likely accompanied by a concurrent decrease in markers of inflammation and oxidative stress.

Glycated haemoglobin (HbA1c), a stable compound categorized as an advanced glycation product (AGE), results from the nonenzymatic bonding of different sugars to haemoglobin (Hb). The level of HbA1c, the most prevalent form generated through this nonenzymatic process, reflects an individual's glycemic status over a span of 2 to 3 months. (Sherwani et al. 2016). Therefore, HbA1c serves as a reliable blood biomarker for diabetes, offering greater dependability compared to blood glucose levels (Koval et al. 2011). Elevated HbA1c values are associated with severe diabetic complications, and reducing HbA1c levels has been shown to decrease morbidity and mortality from diabetes (Khaw et al. 2004; Stratton 2000). The relationship between diabetes mellitus and periodontitis is bidirectional (Graziani et al. 2018; Mikami et al. 2021; Takeda et al. 2021). The findings indicate that lycopene supplementation in NSPT was the most effective treatment for improving HbA1c compared to NSPT alone (Reddy et al. 2015). Propolis, melatonin, GSE, and ginger, similar to lycopene, exhibited a substantial effect size for the intervention. In contrast, the administration of vitamin C did not result in a significant improvement in HbA1c. (Anton et al. 2021; El‐Sharkawy et al. 2016; Gholinezhad et al. 2020).

While HbA1c optimally reflects 2–3 months of glycemic control, we included some studies with shorter follow‐up (4–8 weeks) to maximize evidence synthesis. Our subgroup analysis confirmed the robustness of findings in studies with ≥ 8‐week follow‐up, though this warrants consideration when interpreting results.

Finally, a subgroup analysis for two studies (Anton et al. 2021; Bazyar et al. 2019), revealed significant improvements in PD and CAL when melatonin was used as an adjunct to NSPT. The pooled mean difference for PD was −2.01 (95% CI: −2.52, −1.51; *p* < 0.00001), demonstrating clinically meaningful reductions. CAL also showed significant improvements, further supporting melatonin's efficacy. Moderate heterogeneity (I² = 49%) was observed, likely due to variability in study protocols or patient characteristics, but the consistent direction of effect highlights melatonin's anti‐inflammatory and antioxidant properties as valuable adjuncts to conventional therapy. These findings align with evidence suggesting that adjunctive therapies targeting oxidative stress and inflammation can enhance periodontal outcomes.

## 5 Conclusion

This systematic review and meta‐analysis suggest that the addition of antioxidants, particularly melatonin, to NSPT can lead to statistically and clinically significant improvements in PD and CAL, in patients with T2D and periodontitis, compared to NSPT alone. The findings highlight the potential of adjunctive antioxidants, especially melatonin, to enhance periodontal and systemic outcomes, supported by their anti‐inflammatory and antioxidant properties. Further research is needed to confirm these benefits and refine treatment protocols.

## Author Contributions

*Conceptualization:* Sara A. Abdulla and Hiba Abdelmunim Suliman. *Methodology:* Sara A. Abdulla and Bushra A. Abdalla. *Validation:* Sara A. Abdulla, Hiba Abdelmunim Suliman, and Bushra A. Abdalla. *Formal analysis:* Sara A. Abdulla. *Investigation:* Sara A. Abdulla, Bushra A. Abdalla, Aisha Ali Muhammed, Hayam A. Elawamy, Salima M. Hawda, Enass H. Abduallah, Hajir Omar Alsanfaz, Najah Mohamed, and Mustafa Y. G. Younis. *Data curation:* Bushra A. Abdalla and Aisha Ali Muhammed. *Writing – original draft:* Sara A. Abdulla. *Writing – review and editing:* Sara A. Abdulla, Hiba Abdelmunim Suliman, Aisha Ali Muhammed, Salima M. Hawda, Najah Mohamed, and Mustafa Y. G. Younis. *Visualization:* Bushra A. Abdalla. *Project administration:* Sara A. Abdulla and Bushra A. Abdalla. All authors revised the manuscript and approved the final draft.

## Conflicts of Interest

The authors declare no conflicts of interest.

## Supporting information

Supporting File S1.

Supporting File S2.

Supporting File S3.

## Data Availability

All data obtained in this study are available in the public databases mentioned in the methodology section, and additional details are provided in Supporting Files S1, S2, and S3.
