# Supplementary material for: The Impact of Antioxidant Adjuncts on Periodontal Health in Type 2 Diabetes Patients: A Meta‐Analysis
Source: Clin Exp Dent Res. 2025 Oct 29;11(6):e70215. doi: 10.1002/cre2.70215 (PMC12569448; doi:10.1002/cre2.70215)
Supplement: Supplementary file 1 — Supporting File S1. [file CRE2-11-e70215-s002.docx]

**1.The search terms used in PubMed are listed below:**

The search terms used in PubMed were as follows: “Diabetes Mellitus” or “Glycated Haemoglobin A”), “Antioxidants”), and “Chronic Periodontitis” or “Periodontitis” or “Periodontal debridement”.

(Periodontal diseases [MeSH Terms] OR periodontal disease[Title/Abstract] OR (Chronic Periodontitis [MeSH Terms] OR Chronic Periodontitis [Title/Abstract] OR (Periodontitis [MeSH Terms] OR Periodontitis [Title/Abstract] OR (Periodontal debridement [MeSH Terms] OR Periodontal debridement [Title/Abstract] OR periodontium [MeSH Terms] OR periodontics [MeSH TermsAND (“diabetes mellitus”[MeSH Terms] OR “glycated hemoglobin a”[MeSH Terms] OR “a1c”[Title/Abstract] OR “hb a1c”[Title/Abstract] OR “hba1c”[Title/Abstract] OR “blood glucose”[MeSH Terms] OR “blood sugar”[Title/Abstract] OR ((“glucose”[Title] OR “sugar”[Title]) AND (“level”[Title] OR “control”[Title])) OR “hyperglycemia”[MeSH Terms] OR “hypoglycemia”[MeSH Terms] OR “glycemi*”[Title/Abstract] OR “glycaemi*”[Title/Abstract] OR “hyperglyc*”[Title/Abstract] “hypoglyc*”[Title/Abstract]) AND (“Antioxidants”[MeSH Terms] OR “Antioxidants”[Title/Abstract] OR “antioxidant effect”[Title/Abstract] OR “effect antioxidant”[Title/Abstract] OR “antioxidant effect”[Title/Abstract] OR “antioxidant effect”[Title/Abstract] OR “effect antioxidant”[Title/Abstract] OR “antioxidant effects”[Title/Abstract] OR “antioxidant effects”[Title/Abstract] OR “effects antioxidant”[Title/Abstract] OR “antioxidant effects”[Title/Abstract] OR “effects antioxidant”[Title/Abstract] OR (“melatonin”[MeSH Terms] OR “melatonin”[Title/Abstract] OR “melatonins”[Title/Abstract] OR “melatonine”[Title/Abstract] OR “melatonins” [Title/Abstract]) OR “Ginger”[Title/Abstract] OR “Ginger”[MeSH Terms] OR “O3FAs”[Title/Abstract] OR “O3FAs”[MeSH Terms] OR “ascorbic acid”[MeSH Terms] OR “ascorbic acid”[Title/Abstract] OR “acid ascorbic”[Title/Abstract] OR “l ascorbic acid”[Title/Abstract] OR “acid l ascorbic”[Title/Abstract] OR “l ascorbic acid” [Title/Abstract] OR “vitamin c”[Title/Abstract] OR (“propolis”[MeSH Terms] OR “propolis”[Title/Abstract]) OR (“Lycopene”[MeSH Terms] OR “Lycopene”[Title/Abstract] OR “Lycopene”[All Fields])).

A similar search strategy was applied to all the databases

**2. Embase Search Strategy**

(('periodontitis'/exp OR 'periodontitis':ti,ab OR 'periodontal disease':ti,ab OR 'periodontium':ti,ab) AND ('type 2 diabetes mellitus'/exp OR 'type 2 diabetes':ti,ab OR 'T2DM':ti,ab OR 'diabetes mellitus':ti,ab) AND ('antioxidant agent'/exp OR 'antioxidant*':ti,ab OR 'oxidative stress':ti,ab OR 'free radical scavenger*':ti,ab OR 'melatonin':ti,ab OR 'lycopene':ti,ab OR 'vitamin c':ti,ab OR 'ginger':ti,ab OR 'omega 3 fatty acid*':ti,ab OR 'alpha lipoic acid':ti,ab OR 'propolis':ti,ab) AND ('randomized controlled trial'/exp OR 'clinical trial'/exp OR 'rct':ti,ab OR 'random*':ti,ab))

**3. Scopus Search Strategy**

(TITLE-ABS-KEY("periodontitis" OR "periodontal disease" OR "periodontium") AND

(TITLE-ABS-KEY("type 2 diabetes" OR "T2DM" OR "diabetes mellitus") AND

(TITLE-ABS-KEY("antioxidants" OR "oxidative stress" OR "free radical scavenger*" OR "melatonin" OR "lycopene" OR "vitamin C" OR "ginger" OR "omega-3 fatty acids" OR "alpha-lipoic acid" OR "propolis") AND (TITLE-ABS-KEY("randomized controlled trial" OR "clinical trial" OR "RCT" OR "random*")))

**4. Web of Science Search Strategy**

TS=("periodontitis" OR "periodontal disease" OR "periodontium") AND TS=("type 2 diabetes" OR "T2DM" OR "diabetes mellitus") AND TS=("antioxidants" OR "oxidative stress" OR "free radical scavenger*" OR "melatonin" OR "lycopene" OR "vitamin C" OR "ginger" OR "omega-3 fatty acids" OR "alpha-lipoic acid" OR "propolis") AND TS=("randomized controlled trial" OR "clinical trial" OR "RCT" OR "random*")

**5. Google Scholar Search Strategy**

("periodontitis" OR "periodontal disease" OR "periodontium") AND ("type 2 diabetes" OR "T2DM" OR "diabetes mellitus") AND ("antioxidants" OR "oxidative stress" OR "free radical scavenger" OR "melatonin" OR "lycopene" OR "vitamin C" OR "ginger" OR "omega-3 fatty acids" OR "alpha-lipoic acid" OR "propolis") AND ("randomized controlled trial" OR "clinical trial" OR "RCT")
