# Supplementary material for: The Impact of Antioxidant Adjuncts on Periodontal Health in Type 2 Diabetes Patients: A Meta‐Analysis
Source: Clin Exp Dent Res. 2025 Oct 29;11(6):e70215. doi: 10.1002/cre2.70215 (PMC12569448; doi:10.1002/cre2.70215)
Supplement: Supplementary file 2 — Supporting File S2. [file CRE2-11-e70215-s003.docx]

**Table: GRADE Assessment for Each Outcome**

| **Outcome** | **Certainty of Evidence** | **GRADE Domain** | **Reasons for Downgrading/Justification** |
| --- | --- | --- | --- |
| **HbA1c** | Moderate | Inconsistency | Substantial variability in effect sizes (I² > 50%); e.g., Anton et al. (2021) vs. Kunsongkeit et al. (2019) |
|  |  | Risk of Bias | Variation in baseline HbA1c levels; inconsistent adherence reporting |
| **Clinical Attachment Level (CAL)** | High | — | Consistent improvements across studies; low risk of bias and heterogeneity |
| **Gingival Index (GI)** | Moderate | Inconsistency | Mixed results (e.g., Kunsongkeit et al. vs. El-Sharkawy et al.); variability in GI reduction |
|  |  | Risk of Bias | Differences in NSPT quality and periodontal evaluation methods |
| **Probing Depth (PD)** | High | — | Homogeneous reductions across studies; minimal methodological concerns |
