# Supplementary material for: The Impact of Antioxidant Adjuncts on Periodontal Health in Type 2 Diabetes Patients: A Meta‐Analysis
Source: Clin Exp Dent Res. 2025 Oct 29;11(6):e70215. doi: 10.1002/cre2.70215 (PMC12569448; doi:10.1002/cre2.70215)
Supplement: Supplementary file 3 — Supporting File S3. [file CRE2-11-e70215-s001.docx]

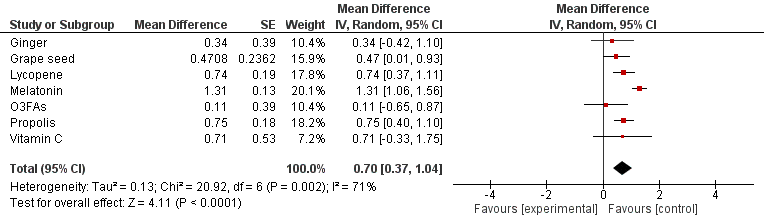


**Figure:** Forest plot showing the performance of different antioxidants used as adjuncts to NSPT compared to NSPT alone on HbA1c.


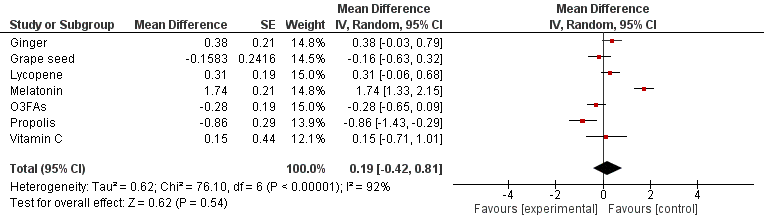


**Figure:** Forest plot showing the effect of different antioxidants used as adjuncts to NSPT compared to that of NSPT alone on CAL.


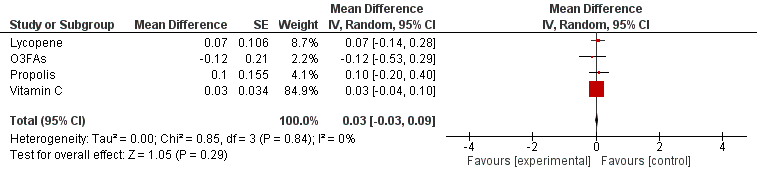


**Figure:** Forest plot showing the performance of different antioxidants used as adjuncts to NSPT compared to NSPT alone on the GI.


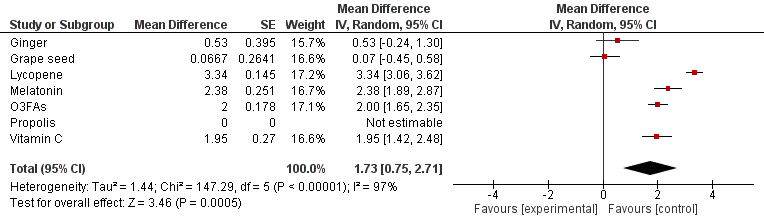


**Figure:** Forest plot showing the performance of different antioxidants used as adjuncts to NSPT compared to NSPT alone on PD.
